# First Report Using a Native Lacewing Species to Control *Tuta absoluta*: From Laboratory Trials to Field Assessment

**DOI:** 10.3390/insects11050286

**Published:** 2020-05-07

**Authors:** Khasan Ismoilov, Minghui Wang, Anvar Jalilov, Xin Zhang, Zhaozhi Lu, Abdusattor Saidov, Xiao Sun, Peng Han

**Affiliations:** 1 CAS Key Laboratory of Biogeography and Bioresource in Arid Land, Chinese Academy of Sciences, Ürümqi 830011, China; khasan_i@163.com (K.I.); wmh2_22@163.com (M.W.); xzhang@ms.xjb.ac.cn (X.Z.); zhaozhi_lv@sina.com (Z.L.); 2 University of Chinese Academy of Sciences, Beijing 100049, China; 3 Institute of Zoology and Parasitology, Academy of Sciences of the Republic of Tajikistan, Dushanbe 734025, Tajikistan; mr.anvar.jalilov@mail.ru (A.J.); abdusattor.s@mail.ru (A.S.); 4 School of Life Sciences, Henan University, Kaifeng 475004, China; sunxiao2017@126.com

**Keywords:** invasive pest, biological control, IPM, exclusion cage, predation rate

## Abstract

The South American tomato pinworm, *Tuta absoluta* (Meyrick) (Lepidoptera: Gelechiidae), a destructive pest on tomato, has invaded most Afro-Eurasian countries. Recently invaded by the pest, most tomato crops in greenhouses and open fields in Tajikistan are currently suffering major damage. While failure in management using chemical insecticide has been frequently observed, alternative options such as biological control is urgently needed. In this study, we evaluated the effectiveness of the common green lacewing *Chrysoperla carnea* (Stephens) (Neuroptera: Chrysopidae) against *T. absoluta*. In controlled laboratory conditions, *C. carnea* showed high predation rate on both *T. absoluta* eggs (i.e., 36 ± 2 eggs within 24 h and 72 ± 4 eggs within 48 h) and larvae, especially it can attack the larvae both inside and outside the leaf galleries (i.e., an average of 22% of the larvae was killed inside, and an average of 35% was killed outside). In a cage exclusion experiment, *T. absoluta* showed relatively low larval density in the cages with pre-fruiting release of *C. carnea*, whereas the larval density was four to six times higher in the “no release” cages. In the “post-fruiting release” cages, the pest population that had already built up during the pre-fruiting stage eventually crashed. In an open-field experiment, the tomato crops in control plots were fully destroyed, whereas low levels of larvae density and damage were observed in the biocontrol plots. Moreover, the field release of *C. carnea* resulted in significantly higher tomato yield than those without release, despite no differences between the “pre-fruiting release” and “post-fruiting release” treatments. We conclude that the local commercial biocontrol agent *C. carnea* could be promising for the management of *T. absoluta* in Tajikistan. It is also one of the first reports showing the management of *T. absoluta* using a lacewing species. The effectiveness should be validated by further field trials in larger area of commercial crops and various locations.

## 1. Introduction

Biological invasions are a major component of global change and are becoming more and more challenging to modern agriculture due to unprecedented increasing trade nowadays [1]. The economic cost due to invasive insect pests has been estimated as 70 billion US dollars per year globally [2]. Management options, such as quarantine procedures, monitoring, eradication, and long-term population suppression, have been developed to reduce their potential economic and environmental impacts. Among the Invasive Alien Species (IAS), insect pests are one of the greatest groups that challenge agricultural and natural ecosystems where Integrated Pest Management (IPM) proves to be a reliable strategy to reduce the harm. To some extent the reliance on the implementation of IPM programs for invasive insect pests is comparable and even greater than those for endemic insect pests, such as chewing and sap-feeding insect pests [3,4,5,6,7,8,9,10,11].

The South American tomato pinworm, *Tuta absoluta* (Meyrick) (Lepidoptera: Gelechiidae), is a destructive pest for solanaceous crops [12,13]. Notably it has become a serious threat to tomato production in both greenhouses and open-fields worldwide [14,15,16,17,18]. Various intrinsic characteristics of this species have made it highly invasive and risky to solanaceous crops including the cryptic nature of larvae, high reproduction potential with multiple overlapping generations, strong dispersal capacity, ability to cope with various abiotic conditions [19,20,21,22,23,24], as well as moderate or high resistance to commonly-used insecticides [12,25]. Its invasion has resulted in decreased yields and quality of fruits, increased control costs, and heavy reliance on chemical insecticides [25], with potential side effects on beneficial arthropods (e.g., through multiple potential sublethal effects [26]), which has disrupted local IPM programs in newly invaded areas [25,27]. By combining preventative and control tactics against *T. absoluta*, new IPM packages need to be built by researchers and growers in vast invaded areas. Among diverse control options, biological control through release of arthropod natural enemies is the most commonly used [28]. Many arthropod species can naturally regulate *T. absoluta* populations in the area of origin of this pest [29,30,31,32,33]. To date, release of arthropod predators has been successfully employed in invaded areas, especially in the Mediterranean basin [28,32]. Parasitoid species such as *Trichogramma* spp. (Hymenoptera: Trichogrammatidae) and *Necremnus* spp. (Hymenoptera: Eulophidae) could also attack the eggs and larvae effectively [34,35]. In particular, two commercially available species, *Nesidiocoris tenuis* (Reuter) (Hemiptera: Miridae) and *Macrolophus pygmaeus* (Rambur) (Hemiptera: Miridae), are the key ones used as biocontrol agents in IPM programs against *T. absoluta.* Both species are widely used basically for two reasons. The first is their high predation rates on *T. absoluta* eggs and young larvae [36,37], and the second is their nature of polyphagy [38,39,40]. The latter scenario greatly increases the chance of establishment of the predators before the young seedlings are infested by the moth. Despite their advantages as key commercial biocontrol agents, the wide use has been impeded by the concern on potential damage to tomato crops. The damage is associated with their zoophytophagous nature. The degree of damage has been related to relative predator-to-prey abundance, with damage increasing at high predator abundances and low prey densities [41]. Other predators, for example, lacewing species, belonging to the genus *Chrysoperla* Steinmann, 1964, have been suggested efficient in controlling *T. absoluta* [28]. Nevertheless, so far not a single study has offered experimental evidence showing how a lacewing species could efficiently prey on the moth.

Recently introduced in central Asia, the pest has caused serious harm to local agriculture [42]. Tomato is an important vegetable crop in central Asia. In this region, around 12,000 hectares are cultivated in Tajikistan where presence of *T. absoluta* was firstly identified in 2016 [43]. Local growers have largely relied on agronomic and chemical options for managing *T. absoluta*, especially the spraying of insecticides. Control failures have been frequently found in both protected and outdoor tomato crops. This case may be linked to insecticide resistance in local *T. absoluta* populations, because introduced strains from earlier-invaded areas may already carry insecticide resistance genes at high frequency, even without local selection in the site of introduction [44]. Therefore, alternative management options such as biological control using arthropod natural enemies are highly appreciated. Fortunately, local commercial biofactories in Tajikistan are able to do mass rearing of three arthropod natural enemies including the common green lacewing *Chrysoperla carnea* (Stephens) (Neuroptera: Chrysopidae), the egg parasitoid *Trichogramma evanescens* (Westwood) (Hymenoptera: Trichogrammatidae), and the larva parasitoid *Habrobracon hebetor* (Say) (Hymenoptera: Braconidae). Notably *C. carnea* could feed on nectar, pollen and aphid honeydew, and they are also active predators of aphids and other small insects. The species can be mass-reared with low cost and it has been widely released into cotton fields (more than 70% of total growing area) for managing aphids and Lepidopteran pests. 

Therefore, the objectives of our study are: (i) to assess the predation rate of *C. carnea* on *T. absoluta* in laboratory conditions, (ii) to assess the field performance of the predator in suppressing *T. absoluta* populations using exclusion cages, and (iii) to quantify how the release of predators protects tomato yields in open field. 

## 2. Material and Methods

### 2.1. Biological Materials

Tomato, *Solanum lycopersicum* L., variety *Navichok*, was used in the experiments. This variety is commonly cultivated in Tajikistan. The seedlings were grown in a climatic room (T = 25 °C, RH = 65% and L:D = 16:8) located in the Institute of Zoology and Parasitology (IZIP), Academy of Science of the Republic of Tajikistan. 

A colony of *T. absoluta* was set up in IZIP by collecting the larvae from a tomato-growing greenhouse in Nurek District (Latitude: 38°23′21.01″ N; Longitude: 69°19′21.79″ E). The colony was reared on potted tomato plants in mesh cages (55 × 55 × 70 cm). 

The lacewing species, *C. carnea*, originated from the biofactory “Kishovarz”, which provides commercial biocontrol agents in northern Tajikistan. *Chrysoperla carnea* eggs were maintained on cotton cloth tapes kept in glass bottles (10 cm diameter and 20 cm height) in a climatic room (T = 25 °C, RH = 65% and L:D = 16:8) located in IZIP. 

### 2.2. Laboratory Predation Trials

**Figure 1:**
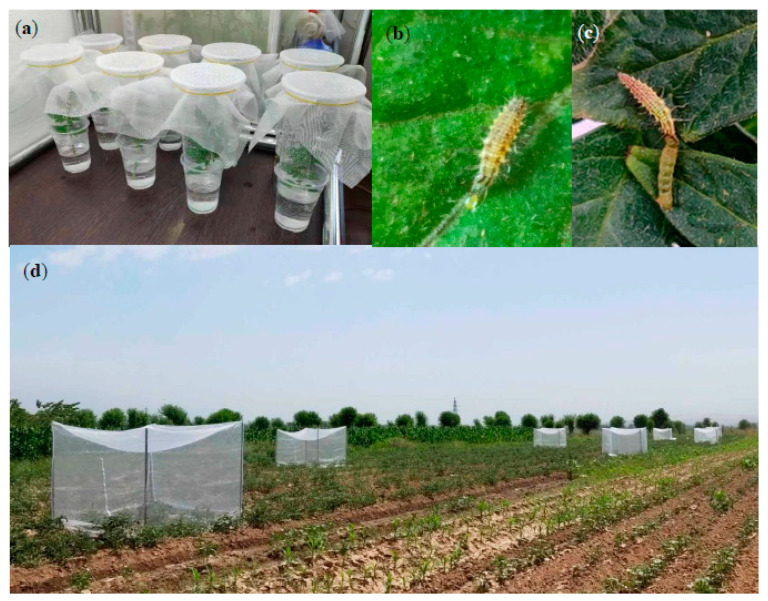
Assessment of *C. carnea* as biocontrol agent against *T. absoluta*: (**a**) double-cup microcosm used in laboratory trials; (**b**) *C. carnea* at third instar larva attacking *T. absoluta* egg; (**c**) *C. carnea* at third instar larva attacking *T. absoluta* larva; (**d**) field assessment on biological control of *C. carnea* against *T. absoluta*: establishment of exclusion cages in tomato field.

*Predation on T. absoluta eggs*: In order to estimate the predation rate of *C. carnea* larvae on *T. absoluta* eggs, *C. carnea* second–third instar larvae and the newly hatched *T. absoluta* eggs (within 12 h) were used in predation trials. The larvae were obtained from the rearing bottles. The eggs were collected from the plant seedlings exposed to the *T. absoluta* colony for 12 h. For the predation bioassay, we used a microcosm design, i.e., double-cup system [45]. Two plastic cups were assembled with the top (600 mL, height: 13 cm) and the bottom one (Figure 1a, 350 mL, height: 11 cm). Leaves were collected from the young shoots of tomato plants and kept individually in the double-cups to preserve leaf turgor. *Tuta absoluta* eggs were transferred onto the leaves by camel brush and one *C. carnea* larva was introduced into each unit. The testing *C. carnea* individuals were starved for 12 h before the bioassay. We set up two treatments for quantifying predation rate. The first was to offer one lacewing 50 eggs and count the eggs consumed after 24 h. The second was to offer one lacewing 100 eggs and count the eggs consumed after 48 h. Twenty and ten replicates were set up for the first and second treatment, respectively. 

*Predation on T. absoluta larvae*: In order to estimate the predation rate of *C. carnea* larvae on *T. absoluta* larvae, *C carnea* second–third instar larvae and *T. absoluta* third instar larvae were used in laboratory trials. To mimic natural conditions, the *T. absoluta* larvae residing inside the feeding galleries were used. The double-up microcosms were set up as described above. One or two *T. absoluta* larvae were used to infest the leaf in each microcosm for 12 h. The microcosm with one *T. absoluta* larva succeeded in residing in feeding gallery was used for the test. Otherwise, the ones with two or none which had succeeded in residing in the feeding gallery were dropped. Twenty microcosms were eventually obtained for the test. Afterwards, one *C. carnea* larva (starved for 12 h) was introduced into each microcosm. After 24 h, the event either “*T. absoluta* larva killed inside the gallery”, “*T. absoluta* larva killed outside the gallery”, or “*T. absoluta* larva not killed” was recorded for each microcosm. The trial was repeated by three times. 

*Tuta absoluta* eggs and larvae survived well within 24 h and 48 h on the leaflets kept under favorable lab conditions. Moreover, we were able to tell the eggs/larvae that had been consumed by the predator from the ones that naturally died. For example, the predator was observed to attack the eggs, suck the liquid content and leave the empty egg shells.

### 2.3. Field Assessment

Field assessment was carried out in two tomato fields in Gissar (Latitude: 38°31′30.14″ N; Longitude: 68°33′4.46″ E) in central Tajikistan (Figure 1d) in 2019. The two fields (0.5 hectares for each) were previously cultivated with tomato with a distance of 500 m. In our experiment, part of each field (around 0.35 ha) was cultivated with tomato seedlings (variety *Navichok*) with the rest of the block being grown with corn. The six-week-old tomato seedlings were transplanted on 27 June. The two fields were cultivated free of insecticide application and only the routine agronomic practices (e.g., tillage, fertilization and irrigation) were applied. The two fields were used for both the cage exclusion experiment and the open-field experiment, as described below. Such a setup was sound since the two independent experiments did not interfere with each other in data collection.

*Cage exclusion experiment*: The cage exclusion technique was used following our previous study [46]. We established 20 cages made of metallic sticks (2 m × 2 m × 2 m) covered by nylon mesh netting with openings of 420 × 420 μm. On 2 August (36 days after transplanting), 12–13 tomato plants were covered in each cage. Subsequently all the plants within each cage were cleaned by removing all insects manually. On 5 August, five pairs of newly emerged *T. absoluta* adults were introduced into each cage. For the 20 cages, we randomly assigned them into three treatments when the crop was in the fruiting stage: (i) seven cages for “pre-fruiting release of *C. carnea*”, i.e., releasing 24 or 26 second instar lacewing larvae (two per plant) into each cage on 14 August (48 days after transplanting); (ii) seven cages for “post-fruiting release of *C. carnea*”, i.e., releasing 24 or 26 second instar lacewing larvae (two per plant) into each cage on 10 September (75 days after transplanting); and (iii) six control cages for “no release of *C. carnea*”. From 14 August to 9 October, numbers of *T. absoluta* young larvae and old larvae and the damage (feeding galleries) on each plant in each cage were recorded. The sampling procedure did not cause any physical damage to the plants. The sampling was conducted every nine to ten days during this period. Tomato yield from three treatments was estimated at the end of the season (i.e., 5–8 October). For each cage, all tomato fruits were collected and weighed to obtain the total yield.

*Open-field experiment*: *Tuta absoluta* occurred naturally in two fields and an average of seven larvae was found in each plant on 14 August. *Chrysoperla carnea* eggs kept on cotton cloth tapes were brought from the biofactory and introduced into one of the fields (i.e., biocontrol field, 0.35 hectare). On 14 August, 29 August (63 days after transplanting), and 25 September (90 days after transplanting), 9000, 12,000, and 13,000 *C. carnea* eggs were released into the biocontrol field. For each release, the releasing points were evenly distributed in the biocontrol field following a “zigzag” pattern. There were around 20 release points. The other field was not released with *C. carnea* eggs (i.e., control field). From 14 August to 9 October, numbers of *T. absoluta* young larvae, old larvae, and the damage (feeding galleries) on each plant in each sampling plot (including 10 plants) were recorded. The plots were chosen by a five-points sampling method. The sampling procedure did not cause any physical damage to the plants. The sampling was conducted every nine or ten days during this period. For the control field, we failed to do the field sampling from 20 September to 9 October. The reason was that the heavy infestation by *T. absoluta* led to heavy damage to the crops, and consequently the plants were almost destroyed around 20 September. 

### 2.4. Data Analyses

We calculated the average number of *T. absoluta* eggs consumed within 24 h (20 replicates) and 48 h (10 replicates). The percentage of the events that the *T. absoluta* larva was killed inside the gallery, outside the gallery, and not killed was calculated for each replicate and the values were averaged (three replicates). For the cage exclusion experiment, we analyzed the effect of the independent variable “*C. carnea* release type” (pre-fruiting release, post-fruiting release, and no release) on the average number of *T. absoluta* young larvae, old larvae, and damage (i.e., feeding gallery) per plant. The average fruit yield per plant from three treatments was calculated and the data were fitted to a one-way ANOVA to check the difference significance. Multiple comparisons were done using a Tukey HSD (honestly significant difference) test. For the open-field experiment, we analyzed the effect of the independent variable “*C. carnea* release” (release vs. control) on the average number of *T. absoluta* young larvae, old larvae, and damage (i.e., feeding gallery) per plant. The data of the cage exclusion experiment and the open-field experiment were fitted in a GEE GLM (Generalized Estimating Equations Generalized Linear Model, ‘geepack’) based on a Poisson error and a log-link function with repeated measurements (‘sampling date’ as repeated factor). For the open-field experiment, only the data from the earlier four sampling dates were used for analyses since the data of the other three sampling dates was not available for the “control”. All the analyses were done using the R software (R Development Core Team, 2009).

## 3. Results

### 3.1. Predation Rate of C. carnea Larvae on T. absoluta Eggs and Larvae

**Figure 2:**
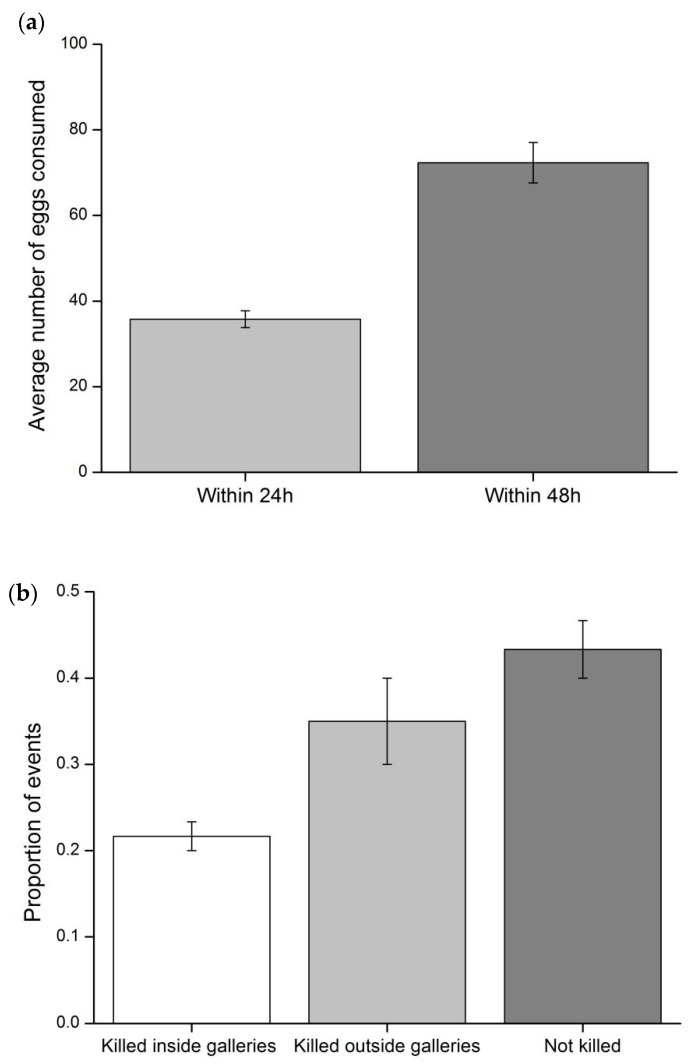
Laboratory microcosm trials of *C. carnea* attacking *T. absoluta*: (**a**) average number (mean ± SE) of *T. absoluta* eggs consumed by each third instar *C. carnea* larva within 24 h (n = 20) and 48 h (n = 10), supplied with 50 eggs and 100 eggs respectively; (**b**) average proportion (mean ± SE) of events that *T. absoluta* larvae were killed inside galleries, outside galleries and not killed by *C. carnea* (n = 3, 20 paired *C. carnea*–*T. absoluta* for each replicate).

*Chrysoperla carnea* larvae were observed to attack *T. absoluta* eggs (Figure 1b). They consumed an average of 36 and 72 eggs within 24 h and 48 h, respectively (Figure 2a). The larva was not only able to attack *T. absoluta* larvae residing inside the gallery, but also able to grab the larvae outside and kill them (Figure 1c, Figure 2b). The average total predation rate was 57% with an average of 22% of *T. absoluta* larvae being killed inside the galleries, and an average of 35% *T. absoluta* larvae being killed outside. 

### 3.2. Field Assessment of C. carnea Controlling T. absoluta

**Figure 3:**
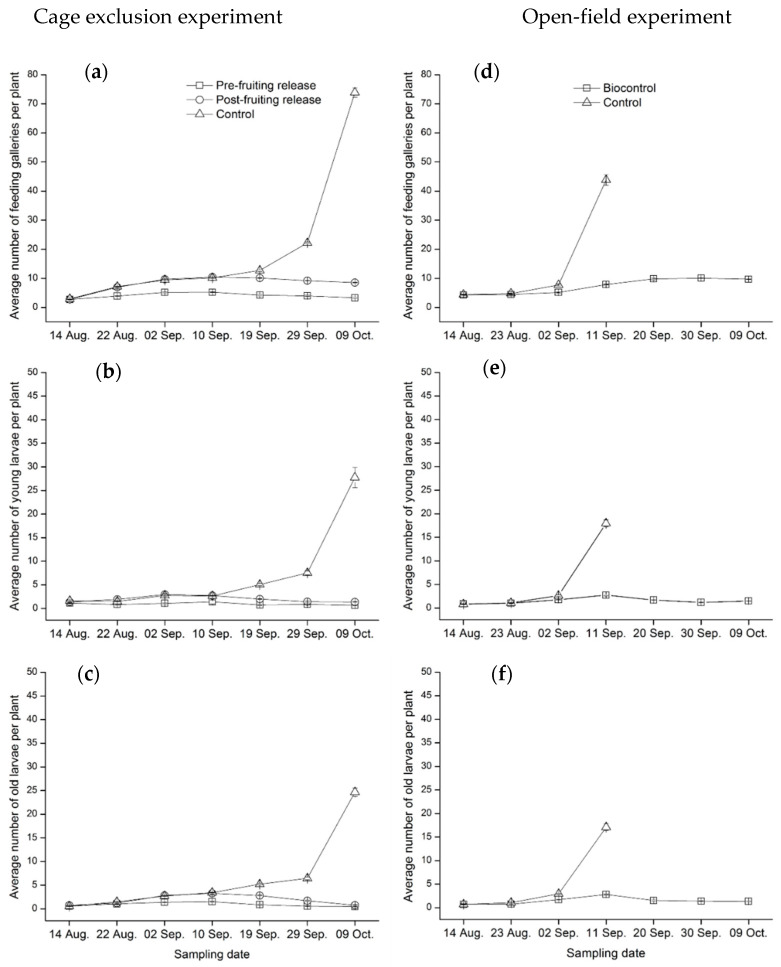
Field assessment of *C. carnea* controlling *T. absoluta*: (**a**) average number (mean ± SE) of feeding galleries per plant, (**b**) average number (mean ± SE) of young larvae (first and second instar) per plant, and (**c**) average number (mean ± SE) of old larvae (third and fourth instar) per plant sampled from the exclusion cages treated with pre-fruiting release of *C. carnea*, post-fruiting release of *C. carnea,* and no release; (**d**) average number (mean ± SE) of feeding galleries per plant, (**e**) average number (mean ± SE) of young larvae (first and second instar) per plant, and (**f**) average number (mean ± SE) of old larvae (third and fourth instar) per plant sampled from the biocontrol field plots and the field without release of *C. carnea* (control).

In the cage exclusion experiment, the number of feeding galleries per plant differed significantly among *C. carnea* release types (χ^2^ = 200.6, df = 2, *p* < 0.001) and among the sampling dates (χ^2^ = 193.4, df = 6, *p* < 0.001) (Figure 3a). The overall variation among dates was mostly attributed to the fluctuation in the control cage (i.e., no release of *C. carnea*). The lowest number of feeding galleries was recorded for the pre-fruiting release cage. The average numbers in both the control cage and the post-fruiting release cage went up slightly and showed a similar trend during the first four sampling dates. However, the two treatments started to show divergence starting on the fifth sampling date. The average number of feeding galleries reached 22 and 75 for the last two sampling dates in the control cage, whereas the numbers maintained below 10 for the post-fruiting release cage. The numbers of young and old larvae per plant differed significantly among *C. carnea* release types (young larvae: χ^2^ = 114.5, df = 2, *p* < 0.001; old larvae: χ^2^ = 125.2, df = 2, *p* < 0.001) and among the sampling dates (young larvae: χ^2^ = 116.8, df = 6, *p* < 0.001; old larvae: χ^2^ = 135.8, df = 6, *p* < 0.001) (Figure 3b,c). The variations among *C. carnea* release types over the sampling dates were similar to those of the feeding galleries. Both young and old larvae were much less abundant in the pre-fruiting and post-fruiting release cages than those of control cages. 

In the open-field experiment, the number of feeding galleries per plant differed significantly among *C. carnea* release types (χ^2^ = 16.00, df = 1, *p* < 0.001) and among sampling dates (χ^2^ = 228.0, df = 3, *p* < 0.001) during the first four sampling dates (Figure 3d). No data was available starting from the fifth sampling date in control plots since all plants were destroyed by the moth. In contrast, relatively low damage level was found in the biocontrol plots until the end of the season. The numbers of young and old larvae per plant differed significantly between two treatments (young larvae: χ^2^ = 17.00, df = 1, *p* < 0.001; old larvae: χ^2^ = 18.00, df = 1, *p* < 0.001) and among the sampling dates (young larvae: χ^2^ = 327.0, df = 3, *p* < 0.001; old larvae: χ^2^ = 430.0, df = 3, *p* < 0.001) (Figure 3e,f). Densities of young and old larvae increased sharply at the fourth sampling date in the control plots, whereas they were much less abundant in the biocontrol plots during the season. 

**Figure 4:**
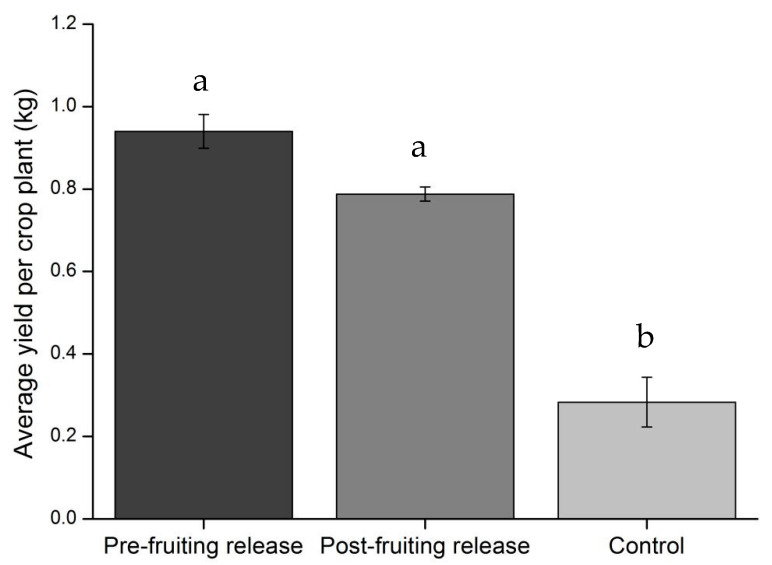
Average yield (in kg, mean ± SE) per crop plant harvested from the exclusion cages treated with pre-fruiting release of *C. carnea*, post-fruiting release of *C. carnea* and no release (control); a,b: values followed by the same letters are not significantly different (*p* > 0.05).

Tomato yield differed significantly among the *C. carnea* release types (*F* = 48.00, df = 2, *p* < 0.001). Cages with pre-fruiting release and post-fruiting release of *C. carnea* resulted in similar yield; however, both treatments led to significantly higher yield than those of control cages (Figure 4). 

## 4. Discussion

This study shows that the lacewing species *C. carnea* is a promising biocontrol agent for *T. absoluta*. *Chrysoperla carnea* was observed preying actively on the moth eggs and larvae in predation trials. Moreover, the release of *C. carnea* during the fruiting stage in exclusion cages and open fields reduced the moth population abundance and protected the tomato yield. As mass rearing of *C. carnea* is not costly in Tajikistan and other central Asian countries, augmentative release of *C. carnea* was suggested to be included in the IPM package against this pest. Our study seems to be the first showing the field evidence that a lacewing species could be used for the management of *T. absoluta*.

Phytophagous mirid predators, such as *N. tenuis* and *M. pygmaeus,* have received greatest interest in the biological control programs against the pest [36,47,48]. *Nesidiocoris tenuis* has been evaluated and considered as a more effective predator since the efficacy of *M. pygmaeus* in controlling *T. absoluta* in the absence of other food sources is possibly limited [48]. Our results showed that *C. carnea* second-third instar larvae were observed to consume around 36 eggs when 50 eggs were offered (Figure 2a), with this predation rate being comparable to those of mirid predators used in Europe [12]. Moreover, it is worth mentioning that *C. carnea* showed strong capacity in consuming the old larvae (e.g., the third instar) of *T. absoluta*, which seemed to be more effective than *N. tenuis* [49]. The tomato crops were almost destroyed by the moth in the control field where no control measure was taken, which prevented us collecting the data during the late season. In contrast, the pest population retained at a stable level in the biocontrol field during the sampling. Releasing *C. carnea* to target *T. absoluta* is supposed to make a great difference to crop yield since tomato crop damage has been primarily attributed to the old larvae of *T. absoluta* [25]. This can be further supported by the field data showing that *C. carnea* release largely increased the tomato yield (Figure 4).

The common green lacewing has the potential to be included in the IPM program targeting *T. absoluta*. Still, several aspects need to be considered for practical use of this species in protected or open-field condition. First, applied aspects of biology and ecology of *C. carnea* need to be further investigated. There are, for instance, the dispersal behavior [50], prey-searching efficiency [51], and functional response to prey densities [52]. For the last point, our data has limitations since we did not offer different densities of eggs to the predator within either 24h or 48h. As a result, we failed to reveal the functional response of *C. carnea* larvae to *T. absoluta* eggs, which may provide valuable knowledge suggesting how many lacewing individuals ought to be released to ensure an effective control of the pest in a given density. Second, predator-mediated indirect interactions among various prey pests may affect the predation pressure of *C. carnea* on *T. absoluta*. *Chrysoperla carnea*, being a generalist predator, may prey on other alternative prey present on the same crop plant. The suppression force on *T. absoluta* may depend on the phenological synchrony exhibited by the co-occurring prey pests [53]. Moreover, in-depth study may be needed to reveal the density- and/or trait-mediated indirect interactions between *T. absoluta* and other key dominate pest species [54]. The knowledge may guide when and how many lacewing individuals should be released into the field. In our field trials, we did an inundative release of *C. carnea* eggs, with two releases during the fruiting stage. Such a release strategy was sound since the pre-fruiting release could perfectly suppress the population build-up. The post-fruiting release was expected to fully protect the fruits from being damaged and guarantee the fruit quality. Lastly, the cost of mass rearing, packaging, transportation, and release need to be assessed. In Tajikistan and other central Asian countries, the cost of mass rearing is low and the commercial release of this species is affordable by local growers. The lacewing products were kept in glass bottles supplemented with alternative Lepidopteran eggs as food. The growers have to pick up the bottled insects from biofactories and return the empty bottles for cyclic use. As *C. carnea* is found in many parts of North America, Europe, and Asia, its usage for controlling *T. absoluta* may be promoted as a key part of the IPM package. Still we should note that the IPM package should not only target *T. absoluta*, as the key pest in tomato crops, but also the other pests in the crop. Our present study has limitations in that only one-year field data was available. However, we intend to show that this biocontrol agent has great potential. The practical use by growers is supposed to yield convincing conclusions in future.

## 5. Conclusions

Our study from laboratory trials to field assessment showed the common green lacewing *C. carnea* could be a promising biocontrol agent for *T. absoluta*. Although conservation of biocontrol agents in the habitat is important [55], mass rearing and augmentative release of *C. carnea* could be a more practical and effective tactic against such a devastating invasive pest. Local biofactories can support mass rearing of this predator, which already lays a solid basis for the promotion of widespread usage, at least in central Asian countries. To enhance its practical usage on larger scale, various applied aspects of the lacewing need to be further investigated. Although *C. carnea* was able to work alone to control *T. absoluta* in the field experiment, we do not suggest the sole use of lacewing by growers. The reason is that alternative treatments may work jointly to suppress *T. absoluta*. For instance, the IPM package could include sex pheromone-based control tools and *Bacillus thuringiensis*-based insecticide.

## References

[1] Paini D.R., Sheppard A.W., Cook D.C., De Barro P.J., Worner S.P., Thomas M.B. (2016). Global threat to agriculture from invasive species. Proc. Natl. Acad. Sci. USA.

[2] Bradshaw C.J.A., Boris L., Bellard C., Roiz D., Albert C. (2016). Massive yet grossly underestimated global costs of invasive insects. Nat. Commun..

[3] Witzgall P., Stelinski L., Gut L., Thomson D. (2008). Codling moth management and chemical ecology. Annu. Rev. Entomol..

[4] Desneux N., Luna M.G., Guillemaud T., Urbaneja A. (2011). The invasive South American tomato pinworm, *Tuta absoluta*, continues to spread in Afro-Eurasia and beyond: The new threat to tomato world production. J. Pest Sci..

[5] Ragsdale D.W., Landis D.A., Brodeur J., Heimpel G.E., Desneux N. (2011). Ecology and management of the soybean Aphid in North America. Ann. Rev. Entomol..

[6] Giorgini M., Guerrieri E., Cascone P., Gontijo L. (2018). Current strategies and future outlook for managing the neotropical tomato pest *Tuta absoluta* (Meyrick) in the Mediterranean basin. Neotrop. Entomol..

[7] Gervassio N.G.S., Luna M.G., Minardi G.M., Sanchez N.E. (2019). Assessing inoculative releases of *Pseudapanteles dignus* (Hymenoptera: Braconidae) for the biological control of *Tuta absoluta* (Lepidoptera: Gelechiidae). Crop Prot..

[8] Manohar T.N., Sharma P.L., Verma S.C., Chandel R.S. (2019). Demographic parameters of the indigenous egg parasitoids, Trichogramma spp., parasitizing the invasive tomato leafminer, *Tuta absoluta* (Meyrick) (Lepidoptera: Gelechiidae). Egypt. J. Biol. Pest Control.

[9] Alikhani M., Safavi S.A., Iranipour S. (2019). Effect of the entomopathogenic fungus, *Metarhizium anisopliae* (Metschnikoff) Sorokin, on demographic fitness of the tomato leaf miner, *Tuta absoluta* (Meyrick) (Lepidoptera: Gelechiidae). Egypt. J. Biol. Pest Control.

[10] Naik S.O., Kannan G.S., Chakravarthy A.K. (2019). Impact of integrated pest management modules on natural enemies of whiteflies, *Bemisia tabaci* (Genn.) in bitter gourd ecosystem. J. Biol. Control.

[11] Sain S.K., Monga D., Kumar R., Nagrale D.T., Hiremani N.S., Kranth S. (2019). Compatibility of entomopathogenic fungi with insecticides and their efficacy for IPM of *Bemisia tabaci* in cotton. J. Pestic. Sci..

[12] Desneux N., Wajnberg E., Wyckhuys K.A.G., Giovanni B., Salvatore A., Consuelo A.N.V., Joel G.C., Diana C.R., Elisabeth T., Jacques F. (2010). Biological invasion of European tomato crops by *Tuta absoluta*: Ecology, geographic expansion and prospects for biological control. J. Pest Sci..

[13] Xian X.Q., Han P., Wang S., Zhang G.F., Liu W.X., Desneux N., Wan F.H. (2017). The potential invasion risk and preventive measures against the tomato leafminer *Tuta absoluta* in China. Entomol. Gen..

[14] Campos M.R., Biondi A., Adiga A., Guedes R.N.C., Desneux N. (2017). From the Western Palaearctic region to beyond: *Tuta absoluta* 10 years after invading Europe. J. Pest Sci..

[15] Sankarganesh E., Firake D.M., Sharma B., Verma V.K., Behere G.T. (2017). Invasion of South American tomato pinworm, *Tuta absoluta* (Meyrick) (Lepidoptera: Gelechidae) in Northeastern India: A new challenge and biosecurity concerns. Entomol. Gen..

[16] Mansour R., Brévault T., Chailleux A., Cherif A., Grissa-Lebdi K., Haddi K., Biondi A. (2018). Occurrence, biology, natural enemies and management of *Tuta absoluta* in Africa. Entomol. Gen..

[17] Han P., Zhang Y.N., Lu Z.Z., Wang S., Biondi A., Desneux N. (2018). Are we ready for the invasion of *Tuta absoluta*? Unanswered key questions for elaborating an Integrated Pest Management package in Xinjiang, China. Entomol. Gen..

[18] Verheggen F., Fontus R.B. (2019). First record of *Tuta absoluta* in Haiti. Entomol. Gen..

[19] Han P., Lavoir A.V., Le Bot J., Amiens-Desneux E., Desneux N. (2014). Nitrogen and water availability to tomato plants triggers bottom-up effects on the leafminer *Tuta absoluta*. Sci. Rep..

[20] Han P., Desneux N., Amiens-Desneux E., Le Bot J., Bearez P., Lavoir A.V. (2016). Does plant cultivar difference modify the bottom-up effects of resource limitation on plant-herbivorous insect interactions?. J. Chem. Ecol..

[21] Sohrabi F., Nooryazdan H., Gharati B., Saeidi Z. (2016). Evaluation of ten tomato cultivars for resistance against tomato leaf miner, *Tuta absoluta* (Meyrick) (Lepidoptera: Gelechiidae) under field infestation conditions. Entomol. Gen..

[22] Blazhevski S., Kalaitzaki A.P., Tsagkarakis A.E. (2018). Impact of nitrogen and potassium fertilization regimes on the biology of the tomato leaf miner *Tuta absoluta*. Entomol. Gen..

[23] Cherif A., Attia-Barhoumi S., Mansour R., Zappalà L., Grissa-Lebdi K. (2019). Elucidating key biological parameters of *Tuta absoluta* on different host plants and under various temperature and relative humidity regimes. Entomol. Gen..

[24] Sylla S., Brevault T., Monticelli L.S., Diarra K., Desneux N. (2019). Geographic variation of host preference by the invasive tomato leaf miner *Tuta absoluta*: Implications for host range expansion. J. Pest Sci..

[25] Biondi A., Guedes R.N.C., Wan F., Desneux N. (2018). Ecology, worldwide spread, and management of the invasive South American tomato pinworm, *Tuta absoluta*: Past, present, and future. Ann. Rev. Entomol..

[26] Desneux N., Decourtye A., Delpuech J.M. (2007). The sublethal effects of pesticides on beneficial arthropods. Ann. Rev. Entomol..

[27] Han P., Desneux N., Becker C., Larbat R., Le Bot J., Zhang J., Lavoir A. (2019). Bottom-up effects of irrigation, fertilization and plant resistance on *Tuta absoluta*: Implications for integrated pest management. J. Pest Sci..

[28] Zappalà L., Biondi A., Alma A., Al-Jboory I.J., Arnò J., Bayram A., Chailleux A., El-Arnaouty A., Gerling D., Guenaoui Y. (2013). Natural enemies of the South American moth, *Tuta absoluta*, in Europe, North Africa and Middle East, and their potential use in pest control strategies. J. Pest Sci..

[29] Miranda M., Picanço M., Zanuncio J., Guedes R. (1998). Ecological life table of *Tuta absoluta* (Meyrick) (Lepidoptera: Gelechiidae). Biocontrol Sci. Technol..

[30] Picanço M.C., Bacci L., Queiroz R.B., Silva G.A., Fabio S., Leite G.L.D., Motta M.M.M. (2011). Social wasp predators of *Tuta absoluta*. Sociobiology.

[31] Bacci L., Silva É.M., Silva G.A., Silva L.J., Rosado J.F., Samuels R.I., Picanco M.C. (2018). Natural mortality factors of tomato leafminer *Tuta absoluta* in open-field tomato crops in the South America. Pest Manag. Sci..

[32] Urbaneja A., Desneux N., Gabarra R., Arnó J., González-Cabrera J., Mafra Neto A., Stoltman L., Pinto A.D.S., Parra J.R.P., Peña J.E. (2013). Biology, ecology and management of the South American tomato pinworm, *Tuta absoluta*. Potential Invasive Pests of Agricultural Crops.

[33] Campos M.R., Monticelli L.S., Béarez P., Amiens-Desneux E., Wang Y.S., Lavoir A.V., Zappalà L., Biondi A., Desneux N. (2020). Impact of a shared sugar food source on biological control of *Tuta absoluta* by the parasitoid *Necremnus tutae*. J. Pest Sci..

[34] Chailleux A., Biondi A., Han P., Tabone E., Desneux N. (2013). Suitability of the Pest–Plant System *Tuta absoluta* (Lepidoptera: Gelechiidae)–Tomato for Trichogramma (Hymenoptera: Trichogrammatidae) Parasitoids and Insights for Biological Control. J. Econ. Entomol..

[35] Gebiola M., Bernardo U., Ribes A., Gibson G.A.P. (2015). An integrative study of *Necremnus Thomson* (Hymenoptera: Eulophidae) associated with invasive pests in Europe and North America: Taxonomic and ecological implications. Zool. J. Linn. Soc. Lond..

[36] Biondi A., Zappalà L., Di Mauro A., Garzia G.T., Russo A., Desneux N., Siscaro G. (2016). Can alternative host plant and prey affect phytophagy and biological control by the zoophytophagous mirid *Nesidiocoris tenuis*?. BioControl.

[37] Sylla S., Brevault T., Streito J.C., Diarra K. (2016). First Record of *Nesidiocoris tenuis* (Reuter) (Heteroptera: Miridae), as a Predator of the Tomato Leaf Miner, *Tuta absoluta* (Meyrick) (Lepidoptera: Gelechiidae), in Senegal. Egypt. J. Biol. Pest Control.

[38] Bompard A., Jaworski C.C., Bearez P., Desneux N. (2013). Sharing a predator: Can an invasive alien pest affect the predation on a local pest?. Popul. Ecol..

[39] Han P., Dong Y.C., Lavoir A.V., Adamowicz S., Bearez P., Wajnberg E., Desneux N. (2015). Effect of plant nitrogen and water status on the foraging behavior and fitness of an omnivorous arthropod. Ecol. Evol..

[40] Jaworski C.C., Bompard A., Genies L., Amiens-Desneux E., Desneux N. (2013). Preference and prey switching in a generalist predator attacking local and invasive alien pests. PLoS ONE.

[41] Castañé C., Arnó J., Gabarra R., Alomar O. (2011). Plant damage to vegetable crops by zoophytophagous mirid predators. Biol. Control.

[42] Han P., Bayram Y., Shaltiel-Harpaz L., Sohrabi F., Saji A., Uulu T.E., Jalilov A., Ali A., Shashank P.R., Ismoilov K. (2019). *Tuta absoluta* continues to disperse in Asia: Damage, ongoing management and future challenges. J. Pest Sci..

[43] Saidov N., Srinivasan R., Mavlyanova R., Qurbonov Z. (2018). First report of invasive South American tomato leaf miner *Tuta absoluta* (Meyrick) (Lepidoptera: Gelechiidae) in Tajikistan. Fla. Entomol..

[44] Guedes R.N.C., Picanço M.C. (2012). The tomato borer *Tuta absoluta* in South America: Pest status, management and insecticide resistance. Bull. OEPP.

[45] Larbat R., Adamowicz S., Robin C., Han P., Desneux N., Le Bot J. (2016). Interrelated responses of tomato plants and the leaf miner *Tuta absoluta* to nitrogen supply. Plant Biol..

[46] Yao Y.S., Han P., Niu C.Y., Dong Y.C., Gao X.W., Cui J.J., Desneux N. (2016). Transgenic Bt cotton does not disrupt the Top-down forces regulating the cotton Aphid in Central China. PLoS ONE.

[47] Chailleux A., Bearez P., Pizzol J., Amiens-Desneux E., Ramirez-Romero R., Desneux N. (2013). Potential for combined use of parasitoids and generalist predators for biological control of the key invasive tomato pest, *Tuta absoluta*. J. Pest Sci..

[48] Mollá O., Biondi A., Alonso-Valiente M., Urbaneja A. (2014). A comparative life history study of two mirid bugs preying on *Tuta absoluta* and *Ephestia kuehniella* eggs on tomato crops: Implications for biological control. BioControl.

[49] Urbaneja A., Montón H., Mollá O. (2009). Suitability of the tomato borer *Tuta absoluta* as prey for *Macrolophus pygmaeus* and *Nesidiocoris tenuis*. J. Appl. Entomol..

[50] Tabone E., Bardon C., Desneux N. (2012). Study of dispersal as a selection criterion for Trichogrammatidae for biological controlin cauliflower greenhouses. Acta Hortic..

[51] Gontijo L.M., Nechols J.R., Margolies D.C., Cloyd R.A. (2012). Plant architecture and prey distribution influence foraging behavior of the predatory mite *Phytoseiulus persimilis* (Acari: Phytoseiidae). Exp. Appl. Acarol..

[52] Madadi H., Parizi E.M., Allahyari H., Enkegaard A. (2011). Assessment of the biological control capability of *Hippodamia variegata* (Col.: Coccinellidae) using functional response experiments. J. Pest Sci..

[53] Desneux N., Kaplan I., Yoo H.J.S., Wang S., O’Neil R.J. (2019). Temporal synchrony mediates the outcome of indirect effects between prey via a shared predator. Entomol. Gen..

[54] Han P., Becker C., Le Bot J., Larbat R., Lavoir A.V., Desneux N. (2019). Plant nutrient supply alters the magnitude of indirect interactions between insect herbivores: From foliar chemistry to community dynamics. J. Ecol..

[55] Gurr G.M., Wratten S.D., Landis D.A., You M.S. (2017). Habitat management to suppress pest populations: Progress and prospects. Annu. Rev. Entomol..

